# Hydrophilic and Hydrophobic Surfaces: Features of Interaction with Liquid Drops

**DOI:** 10.3390/ma16175932

**Published:** 2023-08-30

**Authors:** Dmitrii V. Antonov, Anastasya G. Islamova, Pavel A. Strizhak

**Affiliations:** 1 Heat and Mass Transfer Laboratory, National Research Tomsk Polytechnic University, 30 Lenin Avenue, Tomsk 634050, Russia; dva14@tpu.ru (D.V.A.); agi2@tpu.ru (A.G.I.); 2 A. N. Frumkin Institute of Physical Chemistry and Electrochemistry RAS, Moscow 119071, Russia

**Keywords:** hydrophilic surface, hydrophobic surface, surface modification, drop, interaction, experiment, modeling

## Abstract

The processes of interaction of liquid droplets with solid surfaces have become of interest to many researchers. The achievements of world science should be used for the development of technologies for spray cooling, metal hardening, inkjet printing, anti-icing surfaces, fire extinguishing, fuel spraying, etc. Collisions of drops with surfaces significantly affect the conditions and characteristics of heat transfer. One of the main areas of research into the interaction of drops with solid surfaces is the modification of the latter. Changes in the hydrophilic and hydrophobic properties of surfaces give the materials various functional properties—increased heat transfer, resistance to corrosion and biofouling, anti-icing, etc. This review paper describes methods for obtaining hydrophilic and hydrophobic surfaces. The features of the interaction of liquid droplets with such surfaces are considered. The existing and possible applications of modified surfaces are discussed, as well as topical areas of research.

## 1. Introduction

The interaction processes of liquid drops with solid surfaces determine the characteristics of many technological and natural processes [1,2,3]: inkjet printing, metal hardening, medicine applications, pesticide spraying, coating, spray cooling, etc. The contact time of droplets with the surface has a strong influence on the characteristics of processes in anti-icing technologies [4]. The deposition of thin multilayer films on solid surfaces is used in the production of optics, sensors, etc. [5]. In fluid bed reactors, the wettability of catalyst particles affects the efficiency of the catalyst and the productivity of the process [5]. The conditions and characteristics of water drops’ interaction with pipe surfaces, heaters, and coolers significantly depend on the integral characteristics of heat transfer [6,7]. In recent years, the modernization of solid surfaces through the creation of special super-thin layers has become the most important direction in the development of heat transfer technologies [8,9]. Increasing the interaction area of the liquid with the surface enhances the removal or supply of thermal energy. Special holes, cavities, grooves, artificial elements of porosity, and roughness are used for these purposes [10]. The chemical composition of near-surface layers often change [11,12]. Also, surface modification can be conducted using laser irradiation [13,14,15]. It is possible to achieve fundamentally new properties of surfaces using such techniques. The characteristics of hydrophilic and hydrophobic surfaces can be controlled over a wide range. These surfaces make it possible to solve the complex technological problems of erosion, corrosion, adhesion, and freezing [16,17,18]. Of decisive importance are the disadvantages inherent in superhydrophobic surfaces (for example, reduced values of mechanical strength [19] and chemical resistance of water repellents [20]). It is important to expound unmanned technologies with the development of unpiloted aerial vehicles and the development of the Far North territories [21]. Specialized materials with unique properties are needed. Opportunities arise to eliminate the use of aggressive technological fluids to minimize environmental impact.

The number of articles showing results of experimental studies of the interaction characteristics of liquid drops (water, fuels, emulsions, solutions, suspensions, etc.) with solid surfaces (substrates, grids, rings, particles, etc.) has increased manifold over the past 5–7 years due to the active development of measuring technologies [22,23]. In studies of the interaction between drops and surfaces, materials are used that are characterized by different textures: glass [24], silicon [25], aluminum [15], copper [26], steel [27], etc. The properties of the liquid and the surface are strong influences on the mode and consequences of interaction. Articles [28,29,30] have been published that describe the effect of wetting, liquid properties (for example, viscosity), surface characteristics (roughness, chemical composition), and impact velocity. Much attention is paid to studies of the interaction of drops under conditions of heating [31] and supercooling [4,32]. High-speed video recording, shadow methods, laser diagnostic systems, and microscopic photography are often used to study ultrafast and multiscale interactions of drops with solid surfaces [33,34]. Tracking algorithms for identifying the position of liquid layers in the registration area for processing experimental data have been developed [35]. Unique ideas about secondary fragments (number, size, component composition, separation rates, etc.) formed during the rebound of parent droplets from solid surfaces have been obtained [34].

New knowledge based on experimental results has made it possible to significantly develop physical and mathematical models to describe the processes of liquid drops’ interaction with solid surfaces. To date, there are known approaches for semi-empirical [36,37], numerical, and analytical [38,39,40] solutions to problems stemming from liquid drops’ interaction with solid surfaces. Modern models differ significantly in scale factors. In particular, they are used to study the characteristics of impact with the surface of a single drop [28,29], a certain set of such drops [41], spray [41,42], and film formation [43]. Author’s codes and commercial modeling packages are used. VOF, CFD, etc., methods are used [35].

It is important to perform a comparative analysis of the solutions to the most urgent problems in the selected scientific direction and note promising tasks to solve in the coming years. This motivated an appropriate comparative analysis. Thus, the purpose of this review was to determine the modern achievements in the field of creating hydrophilic and hydrophobic surfaces and to establish the key values of the characteristics of liquid drop interactions with them (wetting, impacts, evaporation, etc.) under experimental and theoretical study. Furthermore, in each section, there are explanations of the most interesting solutions to the urgent problems, as well as problems that have not been fully solved.

## 2. Hydrophilic and Hydrophobic Surfaces

**Figure 1:**
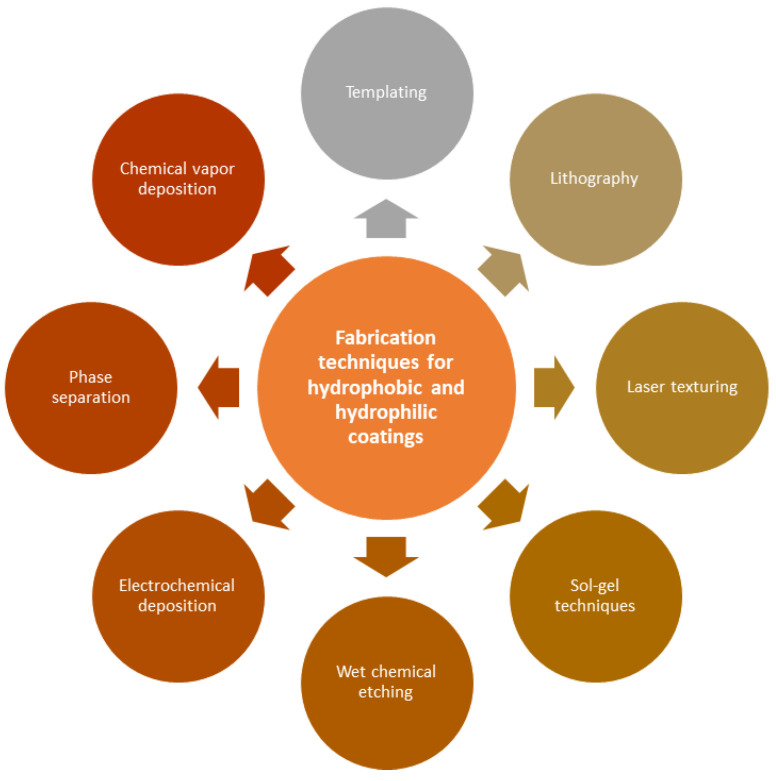
Methods for modifying surface texture.

After modifying the texture and elemental composition using various methods, for example, laser processing [26], layer-by-layer assembly [44], sol-gel techniques [45], chemical etching [46], etc., their wetting properties also can be changed. The most common methods for texture modification, as a result of which wetting properties can be changed, are presented in Figure 1.

### 2.1. Hydrophilic Surfaces

As a rule, there are two ways to make a surface hydrophilic: deposition of a more hydrophilic material film on the surface; change in the chemical composition of the surface layer. At the same time, coating is more common on inorganic surfaces. The chemical modification method is most often used in the case of polymer surfaces.

Sun et al. [47,48,49,50] published interesting research results on self-repairing hydrophilic coatings. The minimum value of the contact angle (θ) during wetting was 53° when applying PVA-Nafion films on the surfaces of glass, silicon, and plastic [47]. It was determined that the film thickness affects the anti-fog and frost-resistant properties of the resulting coatings. Cleaned surfaces of glass and plastic with a pre-deposited poly(diallyldimethylammonium chloride) layer were alternately immersed in HA and bPEI to obtain (HA/bPEI)∗*n* films [48]. After that, the obtained samples were immersed in PFOS solution. When such surfaces were wetted with water, the contact angle was 49°. It has been established that the resulting films have hydrophilic and oil-repellent properties, as well as the ability to self-repair. Howarter and Youngblood [51] obtained brush surfaces of oligomeric amphiphiles of polyethylene glycol with short perfluorinated end caps (f-PEGs) created via grafting methods. The dynamic inflow and outflow wetting angles on such surfaces were 30° and 0°, respectively. When thin films of silica nanoparticles and poly(acrylic acid) were deposited on glass, it was determined that the chemical composition of the surface is the main factor determining the wetting properties of the coatings [24]. It was established that with an increase in the volume concentration of silicon in the solution during coating on glass, the contact angle of wetting with water decreases. It was shown that with an increase in silicon from 53 vol. % to 91 vol. % θ decreases from 10° to 2°. The superhydrophilicity of the coating is due to the presence of hydrophilic functional groups and nanoscale roughness. Dudem et al. [52] obtained hierarchical silver/titanium dioxide/silicon (Ag/TiO_2_/Si) structures with forest-like nano/micro-architectures. These surfaces with water contact angles less than 5° are made using the low-temperature chemical bath deposition technique.

The available methods for obtaining superhydrophilic and oleophobic surfaces include the silanization method. Alkylsiloxane SAM formation is promoted by the presence of silanol groups on the oxide surface, which in turn leads to hydrophilization [53]. It was established [27] that the application of sulfobetaine silane to glass and stainless steel leads to their hydrophilicity (θ < 5°). It was shown that the process of silane film deposition depends on the solvent [54], its concentration, temperature [53,55] and humidity [53,56], deposition time [57], and age of the solution [58]. Failure to comply with the application’s technology can lead to poor adhesion to the modified surface and the formation of an unstable and inhomogeneous film [59]. Parikh et al. [60] set the critical temperature for the alkylsilane deposition. Inhomogeneous and disordered films were formed when this temperature was exceeded. At a low water content, it is more difficult for the octadecyltrichlorosilane molecule to hydrolyze, and the growth of the adsorbed layer may be incomplete [56]. Magnetron sputtering of a titanium oxide film on commercially pure Ti leads to a hydrophilic surface. It was shown [61] that by adjusting the deposition parameters and controlling the modification process, it is possible to vary the wettability of titanium oxide films from 0° to 90°.

### 2.2. Hydrophobic Surfaces

Two approaches are traditionally used to obtain superhydrophobic surfaces. The first approach is to fabricate a surface with micro- and nano-textures on materials with low surface energy. In the second approach, a layer of low surface energy material is deposited on a hard, rough surface (like fluorochemicals and silicones) [62].

Magnetron sputtering of thin films of zinc, titanium oxide, nickel, chromium, silver, etc., is used both to improve and degrade the surface wettability. The contact angle value on the composite surface obtained by magnetron sputtering of nickel on silicon followed by its baking with aluminum powder was 157°, which is almost two times higher than its value on the untreated Si surface [25]. Superhydrophobic nickel films were obtained on SS316L substrates using a combined electrodeposition and fluorination approach [63].

It is known that the surface roughness influences its wetting properties (hydrophobicity and hydrophilicity) [10]. Aligned carbon nanotubes deposited by chemical vapor on the patterned Si template (pillar array) can result in both hydrophilicity and hydrophobicity. Sun et al. [64], by varying the distance between texture elements (pillar), obtained both superhydrophobic (θ = 154.9 ± 1.5°) and hydrophilic (20.8 ± 2.3°) surfaces. Lau et al. [11] deposited conformal hydrophobic poly(tetrafluoroethylene) coating on a silicon surface using aligned carbon nanotubes. The advancing and receding contact angles on such a surface were 170° and 160°, respectively. Emelyanenko et al. [65] deposited methoxy-{3-[(2,2,3,3,4,4,5,5,6,6,7,7,8,8,8-pentadecafluorooctyl)-oxy]-propyl}-silane to the textured surfaces of aluminum–magnesium alloy D16 using the chemical deposition method. As a result, the samples were characterized by a static contact angle of 171.9 ± 0.7° and sliding angle 1.9 ± 0.5°. When exposed to fluorosilane vapors on the magnesium alloy surface, the value of θ reached 171.5 ± 1.0° [66]. The electrodeposition method is used to produce hydrophobic nanostructured layers. This method of fabricating nanostructured layers of zinc oxide has proven to be advantageous for creating one-dimensional and hierarchical ZnO nanostructures [67,68]. Gu et al. obtained stable superhydrophobic films on Ni/Cu [69] and copper alloy [70] substrates using the electrochemical method and obtained superhydrophobic TiO_2_ nanotube coating using anodic oxidation and lauric acid modification [71].

In recent years, texturing methods have become widely used in order to obtain the required functional properties of the surface. Super-hydrophobicity can be achieved by creating a periodic micro/nanostructured texture using laser irradiation and subsequent surface treatment. Laser texturing is classified based on the delivered pulse duration of the laser beam when exposed to the surface of the material [72,73]: femtosecond, picosecond, nanosecond, and millisecond laser texturing. It has been established that after laser texturing, the surfaces exhibit hydrophilic properties. The surface acquires (super)hydrophobic properties during long-term storage in the environment [74,75], low-temperature annealing [76,77], deposition of a hydrophobizator layer [15,78], etc. Table 1 shows examples of superhydrophobic surfaces obtained using laser texturing.

The lithography method is used to create different textures on substrates of various natures. The surfaces obtained in this way have a given thickness and roughness structure. An ordered array of nanopores and nanocapillaries was obtained on the SiO_2_ surface, consisting of columns with sharp vertices using electron beam lithography and plasma etching [83]. The surface exhibited super-hydrophobicity with a contact angle of 164° and a hysteresis of 1° after hydrophobization with octadecyltrichlorosilane. Choi et al. [84] achieved super-hydrophobicity (θ = 164°) using nanoimprint lithography. They produced overhang nanostructures on glass. The disadvantage of this method is that it is only suitable for small surfaces.

Despite the many methods developed to obtain hydrophobic and hydrophilic surfaces, an unsolved problem is their scalability. There is also a task to increase the durability of the resulting coatings (their functional properties). Many surfaces quickly lose their functionality with repeated use.

## 3. Experimental Data on the Interaction of Liquid Droplets with Hydrophilic and Hydrophobic Surfaces

**Figure 2:**
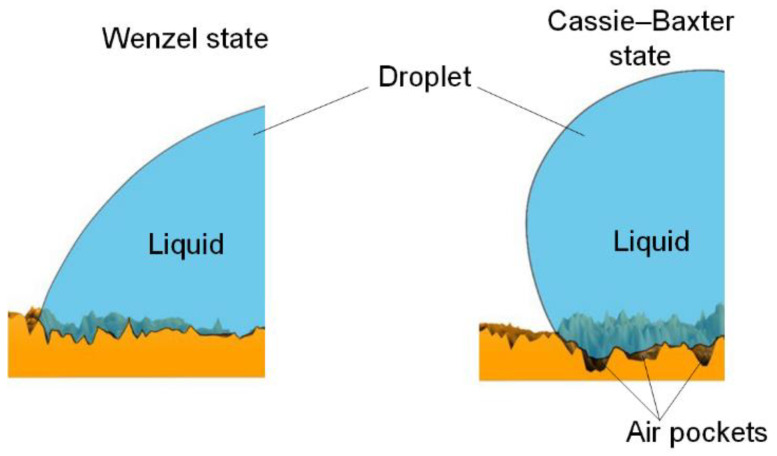
Wetting states. The liquid completely fills the cavities between the surface irregularities in the Wenzel state. Liquid does not penetrate into pockets between surface irregularities (gas remains in them) when the Cassie–Baxter state is realized.

Surface roughness affects its wetting properties and determines the performance and lifespan of the product [85]. The roughness of hard surfaces can be described mathematically using 2D or 3D roughness parameters [86]. The most commonly used parameter to describe roughness is the arithmetical mean deviation of the roughness profile Ra [87]. Boscher et al. [12] used four two-dimensional roughness parameters to describe superhydrophobic surfaces: the arithmetical mean deviation of the roughness profile Ra; the mean height of the roughness Rc; the mean width of the roughness profile elements RSm; and profile elements symmetric height distribution, Rsk. Skilbeck et al. [88] also used two-dimensional parameters: Ra, Rv (valley depth), and Rp (peak height). In mechanical engineering, surface roughness is an important measure of surface quality and is usually described in terms of Ra, Rp, Rv, Rc, Rsk, Rt (total height of the roughness profile), Rq (root mean square roughness), and Rku (kurtosis) [85]. Often, the geometric parameters of pillars (height, width, distance between pillars, etc.) are used when creating ordered pillar structures instead of roughness parameters [89,90,91]. Rahmawan et al. [92] explained the effect of surface roughness on super-hydrophobicity by combining the Wenzel and Cassie–Baxter models for a surface with double micro-nanoscale roughness (Figure 2). It has been found that super-hydrophobicity decreases significantly (from 160° to 130°) when the distance between micropillars exceeds a certain threshold. This effect is due to the fact that with an increase in the distance between micropillars, a transition occurs from the Cassie–Cassie to the Wenzel–Cassie state. It was determined [91] that on the surface of a film containing hexagonal ZnO nanorods, the wettability depends on the length, density, and diameter of ZnO nanorods.

**Figure 3:**
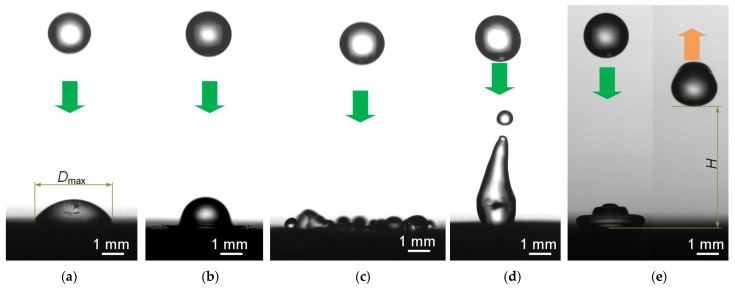
Typical regimes of droplet interaction with super-hydrophobic surfaces: deposition (**a**), splash (**b**), receding break-up (**c**), partial rebound (**d**), and complete rebound (**e**).

The impact of a drop on a solid surface is a complex process. The influence of the collision velocity, droplet size, contact angle, and roughness on the liquid droplets’ collision with a solid surface has been established [28,29]. The following five most frequently occurring regimes can be distinguished when a drop collides with a super-hydrophobic surface (Figure 3): deposition, splash, receding break-up, partial rebound, and complete rebound.

As a rule, dimensionless parameters are used to study the dynamics of the drop interaction with a solid surface: Reynolds number (Re), Weber number (We), capillary number (Ca), and Ohnesorge number (Oh). In addition to dimensionless parameters, quantities such as the maximum spreading coefficient, rebound height, spreading time, icing time are used. Contact time, which can be defined as the time that a falling drop is in contact with a surface before rebounding from it, is also a critical parameter and is closely related to anti-icing, drip condensation, self-cleaning, etc. [4,93]. Super-hydrophobic coatings are used to reduce the interaction time of a drop with a surface [94,95]. The following three characteristic times are used to estimate the drop spreading time: capillary-viscous time τ_v_ [96], advective time τ_a_ [97], and capillary-inertial time τ_ci_ [98]. In this case, the time τ_v_ is applicable for low-viscosity liquids, while τ_a_ and τ_ci_ are used when inertial forces predominate. It was established [98] that the contact time does not depend on We, but is scaled along the inertial-capillary timescale τ_ci_ = √(ρ*R*^3^/σ), where ρ and σ are the density and surface tension of the liquid, and *R* is the radius. At the moment, the universal scaling law describing the spreading time has not been defined.

The maximum spreading coefficient (β_max_ = *D*_max_/*D*_0_, *D*_max_—the maximum spreading diameter, *D*_0_—the initial droplet diameter before collision) is an important technical characteristic in applications such as thermal power engineering, anti-icing technologies, agriculture, etc. The larger the droplet-spreading diameter, the longer the contact time is. The characteristic β_max_ is related to the dimensionless numbers We and Re. Two approaches are most often used to determine *D*_max_/*D*_d_: theoretical [99,100,101], based on the drop energy balance, and empirical [102,103,104]. Significant experimental work has been carried out to study the interaction of single drops with heated surfaces at low Weber numbers (We) [105,106,107]. It has been found that the maximum spreading diameter increases with We [108]. It was found [105] that the droplet spatters with increasing We numbers or at surface temperatures above the Leidenfrost point. It was determined [31] that the collision process does not significantly affect the evaporation time. The authors explain this by the fact that the impact time is much shorter than the evaporation time of the droplet on hot surfaces. Regime cards based on the We number are obtained for the interaction of a single drop with heated surfaces [109,110].

**Figure 4:**
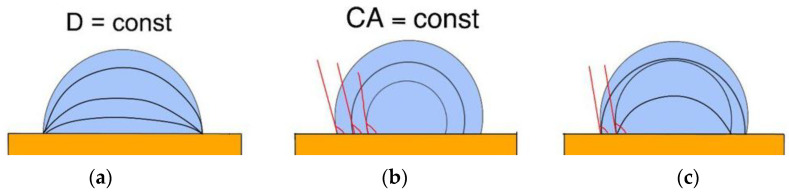
Droplet evaporation modes: pinning (**a**), constant contact angle mode (**b**), and mixed mode (**c**).

Extensive studies have been conducted to study the characteristics of heat transfer during the evaporation of a sessile drop on hydrophobic and hydrophilic surfaces. The following three evaporation modes are distinguished most often during the evaporation of a single sessile drop (Figure 4): pinning mode (contact diameter is constant); constant contact angle mode; and mixed mode (or stick–slide mode). In the pinning mode, the contact line is fixed, and the contact diameter remains constant until the contact angle reaches either 0° or a critical value and the droplet “shrinks”. In constant contact angle mode, the contact line is not fixed. During evaporation, the contact diameter gradually decreases, while the contact angle remains constant. Stick–slide mode alternates between pinning and constant contact angle modes. It was shown [7] that on surfaces with good wettability, the evaporation rate increases due to spreading and convection. The deposition of SiO_2_ and carbon nanotubes on silicon surfaces resulted in an increase in the critical heat flux up to 75.3% compared to the uncoated surface [6]. Microspray cooling on three nano textured surfaces investigated [8]. The conclusion is formulated that surface wettability and liquid spreading are the causes of the heat transfer enhancement [8]. Zaitsev et al. [111] showed that laser treatment of metals is a promising way to obtain surfaces with controlled wetting and boiling characteristics.

A large number of papers are aimed at studying the self-assembly of organic and inorganic particles during the evaporation of droplets on hydrophobic [112,113] and hydrophilic [114,115] surfaces. As a result of self-assembly, both single-layer and multi-layer structures can be formed. The following self-assembly structures are known: crystalline pattern, thin disc, a three-dimensional cap, a circular ring, and multiple rings with different diameters. Changing the wettability properties (roughness and chemical composition) of the surface is one of the ways to control the dried structure morphology.

Basically, there are two modes when droplets collide with solid hydrophilic surfaces [116,117] as follows: deposition-spreading and splashing. Researchers are focused on describing the physical mechanism of droplet splashing and its threshold state when studying the interaction of droplets with surfaces. The traditional empirical model is considered to be the K-parameter model, which takes into account viscous, inertial, and capillary forces [118,119]: *K* = Oh∙Re*^n^*. The empirical factor *n* depends on the viscous forces. For example, at *n* > 1, the fluid viscosity can suppress splashing (*K* = Oh∙Re^5/4^ [118,119]), and at *n* < 1, on the contrary, can promote splashing (*K* = Oh∙Re^0.6089^ [120]). The effect of liquid viscosity on the droplet splashing characteristics after impact of the droplet on a smooth hydrophilic surface has been studied [30]. Regime maps are compiled at the coordinates Re = *f*(Oh) and the interaction rate versus fluid viscosity. It was established [30] that droplet shear viscosity leads to splashing of the droplet under conditions of low viscosity of the liquid, and vice versa; at high viscosity, it prevents splashing. Starinskiy et al. [121] compared the droplet collision with super-hydrophobic (θ = 161°) and super-hydrophilic (θ = 5°) droplets. It was shown that the contact line motion on the surface depends on the type of structure used if its characteristic size is less than 10 μm. Laxman et al. [122] experimentally studied the dynamics of a microliter water drop impact on a hydrophobic microgrooved surface made using photolithography. A regime map = *f*(*p*/*D*_0_) is proposed, where *p*—the distance between microgrooves.

According to classical nucleation theory, the surface curvature, the contact angle between the surface and ice embryos, and the contact angle between droplets and substrate affect ice formation [123]. Davis et al. [124] investigated how surface roughness, skewness, and kurtosis affected surface structure. It was determined that the decrease in ice adhesion is associated with greater hydrophobicity, roughness, skewness, and kurtosis, as well as shorter autocorrelation length [124]. A relationship has been established between the adhesion force and the contact angle hysteresis [125]. It was shown that surfaces with high hysteresis exhibit anti-icing properties due to their structural characteristics [126]. Air in the roughness elements reduces the interaction area of droplets with a super-hydrophobic surface (Figure 2). This increases the time that it takes for the drop to freeze. In addition, the energy barrier for removing water droplets from the super-hydrophobic surface is reduced.

Electrochemical anodization and chemical modification were applied to aluminum surface modification (θ = 155.2 ± 0.5°, roll off angles 3.5 ± 1.3°) [127]. The hydrophobic coating was found to exhibit excellent self-cleaning, anti-icing, and anti-corrosion properties. Thin films of TiN and PTFE were deposited on the surfaces of Q235 steel and silicon using the plasmon-mediated photothermal method [128]. The surfaces were characterized by hydrophobicity (θ ≈ 156°, roll off angles ≈ 2°). It was shown [128] that the freezing time of water on a substrate coated with TiN-PTFE slows down by about 400% times compared to an untreated steel surface (from 305 to 78 s). The coating also showed good thermal stability (up to 200 °C), chemical stability over a wide pH range, corrosion resistance in NaCl solution, and mechanical scratch resistance. Jiang et al. [129] found that the hydrophobic coating of SiC/CNTs made it possible to slow down the freezing time of liquid droplet from 15 to 66 s. Super-hydrophobic surfaces were obtained (θ ≈ 175° and roll off angles ≈ 1.5°) by chemically etching aluminum and coating it with 1H,1H,2H,2H-heptadecafluorodecyl (FD)-trimethoxysilane and poly(dimethylsiloxane) (PDMS)-triethoxysilane characterized by excellent anti-icing properties [130]. It was shown that after 100 icing/melting cycles, the ice adhesion strength was 47.2 kPa. Super-hydrophobic stainless steel surfaces were obtained using nanosecond laser texturing followed by chemisorption of fluorooxysilanes [131]. It was found that the surfaces characterized by weak adhesion to supercooled saltwater drops at −10 °C, and the liquid did not freeze for tens of hours. Wang et al. obtained super-hydrophobic surfaces using laser texturing and low-temperature annealing (θ~159.2°). An excellent anti-icing property is achieved on the fabricated surfaces with water droplets on it retaining the liquid state for over 500 min at −8.5 ± 0.5 °C [132]. Laser-treated aluminum surfaces were characterized by high contact angles (154.3°) and low slide angles (2.1°), while the icing delay time exceeded 700 s [133].

There are still unsolved problems despite the huge number of publications devoted to the study of the interaction of drops with super-hydrophobic/hydrophilic surfaces. In particular, either sessile drops or drops that fall at right angles are mainly considered. There are not enough papers that consider interaction processes when varying the trajectory of falling drops (angle of incidence) on a flat or inclined surface; therefore, additional studies are required. There is no universal scaling law for spreading times and diameters, despite the fact that by now a number of factors and parameters have been identified that affect the collision modes of liquid drops with solid surfaces. The question of the synergistic influence of various factors on the processes of interaction is relevant, for example: the presence of surfactants, polymers, and nanoparticles. In the field of medicine, it is relevant to study the processes of impact of colloidal solutions drops on solid surfaces with different wettability.

## 4. Mathematical Models for Describing the Interaction of Liquid Droplets with Hydrophilic and Hydrophobic Surfaces

**Figure 5:**
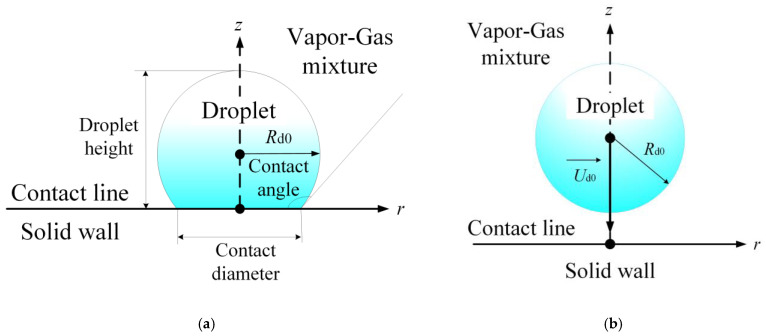
Mathematical models for describing the interaction of liquid drops with solid surfaces: (**a**) sessile droplets; (**b**) falling droplets.

Mathematical models for describing the interaction of liquid droplets with solid surfaces can be divided into two large sections (Figure 5). They include models of sessile droplets on various surfaces [38,39,134,135,136] and dynamic models of droplets falling on solid surfaces [40,137,138,139]. Numerical studies are conducted both on hydrophobic [39,134,136,137,140,141,142] and hydrophilic [143,144] surfaces. For particular cases of super-hydrophilic and super-hydrophobic surfaces, analytical solutions were obtained [145], which play an important role in terms of describing the physics of the process and in the transition to large-scale multiparameter models inherent in a wide class of practical applications (antibacterial/anti-biofouling [146,147], oil recovery [148,149], drag reduction [150,151], anti-corrosion [152,153], self-cleaning [154,155], anti-icing and anti-fogging [156,157], and a combination of both for pool boiling [158,159], oil-water separators [38], lubrication [38], heat pipe wicks [38], dental implants [38], water splitting for environmentally friendly H_2_O_2_ [38] and microfluidics [160]), and natural phenomena (lotus leaves [161], rose petals [162], water ferns [163,164], Sphagnum moss [163,164], water striders [165], butterfly wings [166], etc.).

The importance of the problem of heating and evaporation of sessile drops is well known and widely described [39]. These problems attract the attention of researchers due to a wide range of applications for commercial and industrial use, as well as for the interests of fundamental research [38,39]. The most promising droplet evaporation effects for specific applications can be observed on super-hydrophobic and super-hydrophilic surfaces, which have received considerable attention over the past few decades [38,39]. Modeling of the heating and evaporation processes of sessile droplets has been discussed in numerous works [39,134,140,143]. The authors of papers [39,140] presented reviews of previous models of sessile drop heating and evaporation phenomena, and also proposed their original models. The conjugate problem of heat and mass transfer of droplets on a solid substrate in air was solved numerically [39]. The influence of the boundary layer thickness, surface temperature, wettability, air temperature, and humidity on the processes of heat and mass transfer in evaporating droplets was studied using the volume of fluid method in papers [135,140,167], the lattice Boltzmann method in papers [134,168,169], and molecular dynamic simulation in [141]. In particular, the characteristics of the sessile droplet evaporation under conditions of forced convection [140] were studied. The lifetimes of sessile droplets on heated surfaces [140] were calculated. It was shown that the lifetime of a droplet evaporating under conditions of forced convection is quite accurately predicted by a mathematical model with an error of no more than 8%. The main limitation of the model [140] was the assumption of quasi-stationarity of heat and mass transfer processes in the implementation of direct numerical simulation methods to reduce huge computational costs. The papers [135,136] describe the Marangoni effects in droplets on super-hydrophobic surfaces, including an analytical solution for thermocapillary flow in terms of stream and vorticity functions. A quantitative comparison of the results of modeling and experiments with an estimate of the effective heat transfer coefficients is presented [136]. In paper [141], a model based on molecular dynamic methods was presented and developed to explain the wettability features of a three-dimensional droplet placed on a microtextured surface.

To date, dynamic models of droplet interaction with solid surfaces are quite well developed and allow solving a wide range of problems, including subtle effects of air and gas captured by droplets in contact with solid surfaces [138,170,171], describe the processes of raindrops falling on solid dry and wetted surfaces [172], agricultural problems of leaf treatment with pesticides [173], classical problems of contact line motion [174], etc. In the interests of the development of agricultural technologies, the movement of droplets on leaf surfaces is modeled in order to understand how water and pesticides are absorbed through leaf surfaces. In medicine, the surface of biomaterials plays an important role in determining the results of the interaction between materials and the biological environment [175]. Surface fitting methods (e.g., Clough–Tocher method [176], radial basis functions [177], hybrid method [178]) are used to simulate the process of interaction between water droplets and the leaf surface when constructing a “virtual” surface. Oqielat [179] compared several methods and determined that hybrid multiquadric RBF-CT was the best method for creating a surface in simulation. Research on modeling the processes of the interaction of droplets with surfaces led to the creation of re-entrant topology designs, which are characterized by super-hydrophobicity [180,181].

The result of the interaction of droplets with solid surfaces depends mainly on the impact velocity, the initial droplet size, the properties of the liquid (its density and dynamic viscosity), interfacial tension, the roughness of the solid surface, and its wettability. In general, in the process of droplet interaction with a solid surface, the following modes are numerically implemented: adherence, bouncing, and splashing [182]. In this case, the adherence mode is usually observed with liquid droplets interacting with hydrophilic and super-hydrophilic surfaces, while the bouncing and splashing modes are observed when interacting with hydrophobic and super-hydrophobic surfaces. The main approaches to modeling these processes are based on the methods of volume of fluid [183,184], lattice Boltzmann [170,171], level set [185,186,187], phase field [172,174,188], front tracking [189], diffuse interface model [190], and molecular dynamic simulation [191].

From the point of view of modern numerical modeling, it is most difficult to describe the interaction processes of heterogeneous liquid droplets with micro- and nano-structured surfaces having corresponding functional properties, such as surfaces with complex macroscopic geometry (elongated, concave surfaces, special ribs, etc.). In addition, when comparing the results of numerical simulation obtained using classical methods with experimental data, significant differences are often recorded, which create requirements for the development of new sub-models to describe the processes of heat and mass transfer between a droplet and a solid wall, especially for critical conditions of surface wetting (super-hydrophilic (contact angle less than 10°) and super-hydrophobic (contact angles greater than 150° and hysteresis less than 2°)). In this regard, the most promising modeling problems are the heating and evaporation processes of liquid droplets on structured super-hydrophobic and super-hydrophilic surfaces, considering the complex interrelated processes of heat and mass transfer.

At the moment, in the context of the development of scientific and technological solutions, the unresolved and promising modeling problems are the following: development of a universal model for a droplet and a solid surface interaction; accounting for the irregular droplet shape in contact with textured surfaces; physics of interaction of heterogeneous liquids with surfaces; accounting for the dynamic characteristics of the contact angle/contact line, micro- and nano-roughness of the surface; reduced models of interaction between a droplet and a solid wall for critical wettability conditions.

## 5. Possible Applications of Modified Surfaces

To date, surface phenomena are one of the topical objects of research in medicine, tribology, and liquid chromatography. It was established that the interaction of droplets with surfaces underlies modern technologies for oil and gas displacement from reservoirs, flotation methods for mineral processing, methods for applying paints and coatings, cleaning liquids and gases of impurities, as well as imbuing construction and textile materials with special compositions [94,95,154,155,192]. These characteristics largely determine the rate of formation of nuclei of a new phase and significantly affect the efficiency of heat exchange processes. An urgent problem is the creation of compact drip cooling systems for microelectronic equipment and high-performance processors, the speed and reliability of which depend on the efficiency of dissipated power removal [6,9].

The change in surface tension under the action of surface-active substances (surfactants) is used in washing and laundering, as well as in rock drilling, mechanical processing of high-strength materials, and grinding, causing a significant reduction in energy consumption for these operations. Surface effects are widely used in metallurgy and can change the biocompatibility of various fluids and polymers used in modern medicine for prosthetics [193]. Surface tension is of great importance in biological processes [194]. Many types of composite materials are formed from a liquid dispersion medium (a matrix with a certain viscosity) and a solid dispersion phase (a filler introduced into the system in one way or another). An important condition for the formation of such heterogeneous systems is the optimal ratio between the solid and liquid phases. Many tasks in medicine (for example, the creation of thromboresistant polymers, joint implants, etc.) required a deep study of the wetting of these materials. The peculiarity of the problem lies in the fact that, upon contact with a wetting liquid, the macromolecules of proteins and polymers of the surface layer can gradually change their spatial structure. Upon contact with water, this process leads to a gradual release of polar groups and segments to the interfacial surface. As a result, the interfacial energy decreases with a corresponding change in the contact angle, which in turn can lead to rejection of the material by the body. Extensive work is currently underway to create self-cleaning surfaces [154,155], bioactive surfaces [195], as well as to develop various methods for cleaning bioactive surfaces [196]. Creating an implant surface with desired wetting and bioactive properties is in demand.

Prevention of the icing of aircraft, power lines, and wind turbines by modifying such surfaces is an urgent task [128,197]. Traditional de-icing methods (such as mechanical de-icing, surface heating, de-icing fluids) are inefficient and labor- and energy-intensive. Anti-icing includes slowing down nucleation, increasing freeze time, and reducing ice adhesion. Super-hydrophobic surfaces have large contact angles and a higher potential free energy barrier to nucleation, which contributes to retardation of icing [131,133]. On super-hydrophobic surfaces, the contact area, and hence heat transfer, is much smaller compared to hydrophilic surfaces. Thus, the freezing time of droplets on such surfaces is much longer than on hydrophilic ones [198]. In addition, the small contact area makes it easier to remove frozen droplets. The disadvantages of using super-hydrophobic surfaces in anti-icing technologies include the fact that such surfaces are prone to degradation of wetting (and hence anti-icing) properties during repeated freeze/thaw cycles. At the moment, the creation of super-hydrophobic coatings with good performance properties is topical in this area [130,133].

## 6. Conclusions

(i) To date, several techniques for creating hydrophilic, hydrophobic, and super-hydrophobic surfaces have become widely known. The main ones are described in this review article. Each of the techniques is unique and has both advantages and certain limitations, which are discussed in this article. Using these techniques, it is possible to modify the surfaces of metals, alloys, plastics, and other materials. A generalization of the achievements made it possible to formulate the conclusion that the integral characteristics of surfaces can currently be varied over a wide range.
(ii) This review summarizes the most valuable results of experimental and theoretical studies of the interaction characteristics of liquid droplets with solid surfaces. It has been established that, over the past 10–15 years, a breakthrough has been made in the direction of obtaining new knowledge about the integral characteristics of these processes and the critical conditions for transitions between interaction modes. New experimental data made it possible to significantly develop physical and mathematical models using author’s codes and commercial software packages. The developed experimental and theoretical approaches are tested on hydrophilic, hydrophobic, and super-hydrophobic surfaces when interacting with water, fuels, solutions, suspensions, and emulsions. To date, the integral characteristics have been studied quite fully: the contact spot diameter, the spreading velocity, and the interaction times.
(iii) Energy, transport, petrochemistry, and medicine are the fields for which the research results’ application for studying the collisions with hydrophilic, hydrophobic, and superhydrophobic surfaces are most in demand. By modifying the heat exchanger surfaces, reactors, mixers, transport channels, and other elements, it is possible to change the characteristics of technological processes in an extremely wide range. The most challenging and still unresolved tasks are obtaining stable and durable coatings for wide application in industry, scalability of methods for obtaining surfaces with the desired functional properties, and the synergistic effect of various factors on interaction processes.

## Figures and Tables

**Table 1:** Surface treatment using laser texturing.

Ref.	Material	Treatment Method	Angle
[14]	Silicon	Femtosecond laser texturing (pulse width of 30 fs; wavelength 800 nm; frequency 1 kHz; scanning speed 3 mm/s), layer of fluoroalkylsilane	θ = 160°
[15]	Aluminum alloy	Nanosecond laser texturing (pulse width of 50 ns; peak power of 0.95 mJ; frequency 20 kHz; scanning speed 50 mm/s) and chemisorption	θ ≈ 170° roll off angles 2.1°
[79]	Aluminum alloy	Nanosecond laser texturing (pulse duration 120 ns; pulse frequency, 40 kHz; laser output power 100 W; laser beam velocity 1000 mm/s; scanning density 150 lines/mm; peak fluence 140 J/cm^2^) and fluorosilane deposition	θ = 171.5 ± 0.8° roll off angles 2.0 ± 0.6°
[26]	Copper and polydimethylsiloxane	Femtosecond laser texturing (laser fluence 4–100 J/cm^2^; wavelength 800 nm; frequency 1 kHz; scanning speed 15 mm/s)	θ = 165° roll off angles < 5°
[78]	Tungsten	Nanosecond laser texturing (scanning speed 500 mm/s; scanning line density 25 mm^−1^; number of laser passes 5; effective fluence 715 J/cm^2^)	θ = 172.2 ± 0.5° roll off angles 3.5 ± 1.2°
[80]	Stainless steel	Nanosecond laser texturing (pulse duration 50 ns; pulse frequency 20 kHz; peak pulse power 0.95 mJ; linear rate 50 mm/s; scanning density 150 lines per mm) and chemisorption of a hydrophobic agent CF_3_(CF_2_)_6_(CH_2_)O(CH_2_)_2_C(OCH_3_)_3_	Liquid—polydimethylsiloxane θ = 165 ± 2° roll off angles 3 ± 1°
[81]	Stainless steel AISI 316L	Nanosecond laser texturing (pulse width of 100 ns; peak power of 1 MJ; frequency 200 kHz; scanning speed 200 mm/min) and silanization	θ ≈ 153.2°
[82]	Polydimethylsiloxane	Femtosecond laser texturing (pulse width of 50 fs; wavelength 800 nm; frequency 1 kHz; scanning speed 5 mm/s, power 40 MW) followed by oxygen plasma treatment	After laser treatment θ = 155.5° ± 1.5° After treatment with oxygen plasma θ = 4.5° ± 0.5°

## Data Availability

No new data were created.
